# Clinical-Pathological Conference Series from the Medical University of Graz: Case No 172: A 45-year-old truck driver with fever, vomiting, thrombocytopenia and renal failure

**DOI:** 10.1007/s00508-021-01921-z

**Published:** 2021-08-17

**Authors:** Philipp K. Bauer, Robert Krause, Elisabeth Fabian, Marja-Liisa Aumüller, Dietmar Schiller, Gabriel Adelsmayr, Michael Fuchsjäger, Ernst Rechberger, Rainer Schöfl, Guenter J. Krejs

**Affiliations:** 1 Division of Infectious Diseases and Tropical Medicine, Department of Internal Medicine I, Medical University of Vienna, Vienna, Austria; 2 Division of Infectious Diseases and Tropical Medicine, Department of Internal Medicine, Medical University of Graz, Graz, Austria; 3 Division of Gastroenterology and Hepatology, Department of Internal Medicine III, Medical University of Vienna, Vienna, Austria; 4 Department of Internal Medicine I, Barmherzige Schwestern Hospital, Ried, Austria; 5 Department of Internal Medicine IV, Elisabethinen Hospital, Linz, Austria; 6 Division of General Radiology, Department of Radiology, Medical University of Graz, Graz, Austria; 7 Division of Gastroenterology and Hepatology, Department of Internal Medicine, Medical University of Graz, Auenbruggerplatz 15, 8036 Graz, Austria

**Keywords:** Hantavirus, Hypophysitis, Pseudoacute abdomen, Capillary leak, Nephropathia epidemica

## Presentation of case

### *Dr. D. Schiller*:

The patient is a previously healthy 45-year-old truck driver from Upper Austria. Five days before Christmas he complained of fever up to 39.0 °C, epigastric pain and vomiting. He denied myalgia, headache, vertigo, hematochezia, melena, red-colored urine, dyspnea, hemoptea and chest pain. The patient’s history was negative for drug abuse and travel abroad. Except for the patient, nobody in his community was affected by the described condition. On admission, the patient presented with fever (38.5 °C), a blood pressure of 100/50 mm Hg and an oxygen saturation of 95% at ambient air. Physical examination revealed moderate epigastric pain on palpation but it was otherwise unremarkable (including a cursory neurological examination and inspection of the skin and mucous membranes).

Laboratory data on admission: Hemoglobin 21.5 g/dL (normal: 12.0–15.3 g/dL), leukocytes 23.9 G/L (normal: 4.4–11.3 G/L) with left shift, platelets 31 G/L (normal: 140–440 G/L), C‑reactive protein (CRP) 10.7 mg/dL (normal: < 0.5 mg/dL), D‑dimer 5.26 µg/mL (normal: < 0.50 µg/mL), serum sodium 125 mmol/L (normal: 135–145 mmol/L), aspartate aminotransferase (AST) 55 U/L (normal: 5–34 U/L), alanine aminotransferase (ALT) 66 U/L (normal: < 55 U/L), gamma glutamyl transferase (GGT) 125 U/L (normal: 12–64 U/L), lactate dehydrogenase (LDH) 369 U/L (normal: 125–220 U/L), creatinine 2.09 mg/dL (normal: 0.73–1.18 mg/dL) and lactate 7.02 mmol/L (normal: 0.50–2.20 mmol/L). Urinalysis revealed 250 erythrocytes/µL and 75 mg protein/dL. Serum values for glucose, amylase, lipase, thyroid-stimulating hormone (TSH), free triiodothyronine (fT3), free thyroxine (fT4), potassium, bilirubin, alkaline phosphatase, prothrombin time and fibrinogen were all normal.

Sonography and computed tomography (CT) of the abdomen showed mildly dilated intrahepatic bile ducts, moderate amounts of retroperitoneal fluid, a thickened wall of the second portion of the duodenum and a peripapillary duodenal diverticulum. Chest CT showed bilateral extensive pleural effusions with a pronounced compression atelectasis of the lower lobes. The consulting surgeon suspected a perforated duodenal diverticulum, which constituted an indication for exploratory laparotomy. Intraoperatively, the duodenum appeared unremarkable; however, the gallbladder showed hydrops and there was evidence of peripancreatic edema. An incidental cholecystectomy was performed. Postoperatively, the patient deteriorated and needed hemofiltration and hemodynamic support. On the fourth postoperative day he developed delirium with dystonia, hyperkinetic movements (hemiballism), tonic ocular movements on the left upper side and photophobia. A brain CT, electroencephalogram and laboratory analysis of the cerebrospinal fluid were unremarkable; magnetic resonance imaging (MRI) of the cerebellum revealed significant inhomogeneity of the anterior pituitary with partially missing enhancement, suggesting apoplexy of the pituitary or a cystic macroadenoma. By that time TSH, fT3 and fT4 decreased to levels below normal.

A diagnostic test result became available.

## Differential diagnosis

### *Dr. R. Krause*:

The patient under discussion is a young, previously healthy truck driver who complained of fever, epigastric pain and vomiting. The onset of symptoms was acute and occurred in the month of December. Whenever an infectious disease is suspected, it is vital to pay attention to the season because different infectious risks are associated with different times of the year. The patient’s history was negative for foreign travel and nobody in his community suffered from the same symptoms, which is an important fact that automatically excludes specific infectious diseases typically occurring in clusters. The physical examination was unremarkable, except for fever, low blood pressure and epigastric pain on palpation. Laboratory data revealed leukocytosis with a left shift and thrombocytopenia, and showed increased levels of hemoglobin, CRP, D‑dimer, LDH, lactate and renal function parameters; urinalysis demonstrated hematuria and proteinuria. Abdominal sonography and CT scan showed minimal ascites in the upper abdomen, a thickened wall of the second portion of the duodenum with suspected peripapillary duodenal diverticulum and compromised basal lung parenchyma with bilateral pleural effusion. Since the clinical course worsened, acute abdomen was suspected and an exploratory laparotomy was performed, which, however, yielded unremarkable findings of the duodenum. Thus, the patient may have had a so-called pseudoacute abdomen mimicking acute abdomen. In 1958, Henry Bockus wrote a now classic paper entitled “The internist looks at the acute abdomen” [1], in which several diagnoses causing pseudoacute abdomen are summarized (Table 1).

**Table 1:** Causes of pseudoacute abdomen [1, 44]

Acute intermittent porphyria
Diabetic pseudoperitonitis
Sickle cell crisis
Chronic lead poisoning
Addison’s disease
Familial Mediterranean fever
Spontaneous bacterial peritonitis
Infectious diseases (thyphoid fever, hantavirus, malaria, leptospirosis)
Vasculitis (Henoch-Schönlein purpura, periarteritis nodosa)
Hereditary angioedema
Acute glaucoma
Proptosis
Abdominal epilepsia
Myocardial infarction, acute pericarditis
Dissection of the abdominal aorta
Basal pneumonia, lung infarction, pleurodynia
Hematoma of the abdominal wall (hematoma of the rectus muscle due to anticoagulation)
Herpes zoster

Among others, some infectious diseases, such as leptospirosis, malaria, hantavirus infection or herpes zoster can cause this condition. Since the discussed patient had not been abroad, an autochthonous infection may be the underlying cause of his condition. Postoperatively, the clinical course worsened and the patient developed delirium with dystonia, hyperkinetic movements (hemiballism) and photophobia, which is a symptom that might be pivotal for the diagnosis in this case. The MRI revealed significant inhomogeneity of the anterior pituitary suggesting apoplexy or a cystic macroadenoma.

Fever and elevated CRP levels strongly suggest an infection that is affecting several organ systems, such as bacterial sepsis or a systemic viral infection. Thrombocytopenia is another hallmark, which in combination with fever typically occurs in sepsis caused by infection with bacteria such as *Staphylococcus aureus, Escherichia coli*, enterococci or* Pseudomonas aeruginosa* [2, 3]. For the diagnosis of sepsis, criteria of the sequential (sepsis-related) organ failure assessment (SOFA) score [2] should be employed. This score takes into account sepsis-related alterations of various organ systems including respiration, coagulation, cardiovascular, renal and liver function, and of the central nervous system. In the discussed patient, consideration of these criteria results in a SOFA score of 6 (low blood pressure/mean arterial pressure, thrombocytopenia and renal failure), which confirms the diagnosis of sepsis. As in this case, significantly elevated serum lactate levels are also typically found in septic patients; these may result from tissue hypoxia, a disturbed lactate metabolism caused by renal or hepatic failure, or a combination of these pathophysiologic alterations.

Sepsis affecting multiple organ systems caused by bacteria such as *Escherichia coli, *enterococci or *Pseudomonas aeruginosa* seems unlikely in a previously healthy young man but *Staphylococcus aureus* may cause sepsis in any age group. Further, specific pathogens causing sepsis and thrombocytopenia have to be considered. These include Dengue virus, human immunodeficiency virus, hepatitis C virus, parvovirus B19, hantavirus, *Leptospira spp*. and tick-borne bacteria such as *Rickettsia, Ehrlichia, Anaplasma* and *Borrelia* [4]. Brucellosis is typically associated with thrombocytopenia; however, this disease presents with a slow clinical course and usually does not lead to sepsis [4]. Malaria and leishmaniasis are also diseases characterized by thrombocytopenia, but due to the negative travel history these diagnoses seem unlikely in this case. Suggesting an autochthonous infectious disease in our patient, resulting in sepsis and thrombocytopenia, finally limits the differential diagnosis list to only two candidates: leptospirosis and hantavirus infection.

Renal failure may have been the result of hypotension during sepsis or it may have been caused by direct bacterial damage as is frequently found in leptospirosis. Once in the body, *Leptospira* spread hematogenously, adhere to the host tissue and invade it [5]; they are retained within the renal tubules of hosts, where they thrive and multiply, and are occasionally shed via urine [6]. Leptospirosis presents with protean manifestations, which presumably result from infection with different *Leptospira spp.* inducing different cellular and molecular mechanisms consequently leading to various clinical symptoms. The symptoms of acute phase leptospirosis include sudden onset of fever, myalgia and conjunctival suffusion; nausea, diarrhea, vomiting and chills may also be present [7]. The great majority of infections are either subclinical or mild and run a self-limiting anicteric course [8]. In about 10% of affected patients, infection leads to severe and possibly fatal Weil’s disease, which is characterized by bleeding, renal failure and jaundice caused by intrahepatic cholestasis and direct hepatocyte damage [9, 10]. The main route of acquiring leptospirosis is through mucosal membranes or percutaneous invasion by *Leptospira* excreted by carriers into the environment (e.g. urine in water, soil or other contaminated material). A high incidence has been reported among people who are exposed to wet environments during occupational activities [8] and in those who are exposed to contaminated water during leisure activities such as swimming, canoeing, rafting, fishing and similar sports [8, 11, 12]. Gardening may be an underestimated category of risk exposure in the western world [8]; however, such activities have not been documented in the discussed patient. Direct transmission of *Leptospira* from animals to humans is common among groups who handle animals and animal tissue such as butchers, veterinarians, and cattle and pig farmers [13]. Although some of the clinical features observed in the discussed patient are compatible with the diagnosis of leptospirosis, this disease seems unlikely in this case because of lacking myalgia, which is reported in up to 100% of affected patients [9].

Pathogens other than *Leptospira spp.* that are linked to renal failure include hantavirus, *Treponema pallidum*, cytomegalovirus, Epstein-Barr virus, human immunodeficiency virus and hepatitis virus B and C [4]. Infection with polyomavirus or adenovirus can also cause renal failure but is primarily found in immunocompromised patients; thus, these pathogens can most likely be ruled out as the underlying cause of infection in this case.

Focusing on the clinical feature of sudden onset of photophobia, the following three etiologies have to be considered: (1) ocular causes, (2) affected central nervous system (e.g. meningitis, encephalitis, neuritis) and (3) other causes such as some systemic infections or intoxication (e.g. alcohol intoxication, postoperatively due to narcotics) [14]. In the context of infectious diseases, photophobia may occur due to direct disease involvement of the eye. For example, uveitis with hyphema has been described in a patient with leptospirosis [15] and in another case hypopyon with endophthalmitis was found in infection with *Listeria monocytogenes* [16]. Since no ocular alterations were documented in the discussed patient, infection with these bacteria seems unlikely in this case.

The finding of an abnormal pituitary in our patient and the sudden onset of neurologic symptoms and hyponatremia suggests the following three possible underlying causes: Adenoma of the pituitary, hypophysitis or apoplexy (ischemic or hemorrhagic). Since adenoma of the pituitary developing acutely with a sudden onset of neurologic symptoms is very unlikely, this disease entity can be ruled out definitely. Assuming infection as the underlying cause of hypophysitis various pathogens have to be considered, such as hantavirus, *Aspergillus, Toxoplasma gondii, Blastomyces dermatitidis* and *Histoplasma capsulatum*. Indeed, aspergillosis can most likely be excluded as a differential diagnosis in this case because the infection is primarily found in immunocompromised patients. In immunocompetent individuals, the disease occurs only rarely and predominantly affects subjects who are additionally predisposed by concomitant cerebrovascular disease [17]. Central nervous aspergillosis can be classified into parenchymal lesions and meningeal lesions, which are more frequently associated with cerebral infection-related aneurysms or vascular stenosis, cerebral infarction and subarachnoid hemorrhage [17]. The underlying mechanism of *Aspergillus* vascular invasion is characterized by pathologic alterations caused by inflammation and destruction of vessels [18, 19]. Rarely, infection may also predispose to apoplexy secondary to thrombocytopenia, which has been reported for both septic conditions and infection with hantavirus [20].

Toxoplasmosis seems unlikely in this case because it usually does not present with a fulminant course in an immunocompetent young adult [4]. Due to the negative travel history in our patient histoplasmosis and blastomycosis can also be ruled out as differential diagnosis. Histoplasmosis is caused by the fungus *Histoplasma capsulatum,* which lives in the soil and is endemic in Central and South America, Africa, Asia and Australia [21]. Blastomycosis is a fungal infection caused by inhaled *Blastomyces dermatitidis* spores; it is endemic in the eastern United States and some parts of Canada [22].

Finally, the most likely candidate pathogen that causes abdominal pain (pseudoacute abdomen), an abnormal pituitary due to inflammation or apoplexy, photophobia, and that is further associated with thrombocytopenia and impaired renal function is hantavirus. Hantaviruses are human pathogens which belong to the genus *Hantavirus*, family *Bunyaviridae,* and are found worldwide; numerous genotypes or serotypes have been identified (e.g. Puumala, Hantaan, Dobrava-Belgrade, Seoul, Sin Nombre) [23]. In Western and Central Europe, the majority of hantavirus cases are caused by the Puumala serotype. The Puumala virus is spread by rodents and is transmitted to humans by inhalation of the virus from aerosolized excreta or by ingestion of contaminated food [24]. In Austria, the risk of infection is associated with the natural habitat of the bank vole (*Clethrionomys glareolus*), the reservoir for Puumala virus [25]. Puumala virus infection causes nephropathia epidemica, a certain type of hemorrhagic fever with renal failure [26]. Besides fever which is present in 90–100% of affected patients, abdominal pain (30–43%) and vomiting (26–46%) are key clinical features of this infection [27–30]. Moreover, 23–36% of patients with Puumala virus infection complain of visual changes [28, 30]. In infected children, pseudoacute abdomen (100%) and blurred vision (47%) are even more frequently found than in adult patients [31]. Transient ocular manifestations such as blurred vision (78%), myopic shift (50%), conjunctival injection (22–78% [26, 32]) or subconjunctival hemorrhage (6%) occur in a remarkable portion of infected patients, but are independent of disease severity [26]. The underlying pathophysiologic mechanisms comprise direct ocular effects but may also involve the central nervous system. Direct effects include shallowing of the anterior chamber (which is pathognomonic for Puumala virus infection), thickening of the lens, changes in osmolarity of the lens and aqueous humor, alteration in length of the vitreous cavity and increased tissue permeability [26]. Effects of Puumala virus infection on the central nervous system [20, 32–34] may result in acute disseminated encephalomyelitis affecting variable parts of the brain [24]. Thus, involvement of the parieto-occipital region or the optic nerve may contribute to the ophthalmic manifestations observed during infection. Rarely, concomitant involvement of the pituitary gland causing transient hypopituitarism due to hemorrhage has been reported in Puumala virus infection [20, 32, 33].

In summary, the patient’s signs and symptoms (pseudoacute abomen, involvement of the pituitary, photophobia, renal failure, and increased inflammation markers and thrombocytopenia) strongly suggest hantavirus, most likely Puumala virus infection in this case. In Austria, the risk for infection is highest in certain areas in Styria and Carinthia where *Clethrionomys glareolus*, the reservoir of Puumala virus, is prevalent; however, isolated cases have also been reported in other states of Austria [35]. Besides Puumala, serotypes such as Dobrava-Belgrade and Saaremaa are encountered in Austria. Depending on the serotype, different clinical courses and mortality rates are found (about 1% in Puumala virus infection and 10% in Dobrava-Belgrade virus infection) [36].

Pulmonary manifestations such as inflammatory infiltrates and pleural effusion, as observed in the discussed patient, have been reported in about 53% of cases with Puumala virus infection [37].

The significantly elevated hemoglobin level in this case may suggest absolute erythrocytosis caused by, for example, chronic diseases such as heart failure or chronic obstructive pulmonary disease (COPD), hemoglobin anomalies, abuse of erythropoietin or polycythemia, or relative erythrocytemia due to conditions such as polyuria, exsiccosis caused by increased fever-induced perspiration, or capillary leak which is frequently found in systemic infections (e.g. malaria, leptospirosis, hantavirus infection).

Since Puumala virus infection typically occurs in the spring and summer [38], the presentation of this patient during winter questions the diagnosis but does not rule it out.

In view of the entire constellation of findings in this case, hantavirus infection (probably serotype Puumala) seems to be the most likely diagnosis. This should be confirmed by antibody and PCR tests from blood or tissue.

### *Dr. R. Krause’s diagnosis*

Hantavirus infection (most likely serotype Puumala)

## Discussion of case

### *Drs. G. Adelsmayr and M. Fuchsjäger*:

**Fig. 1:**
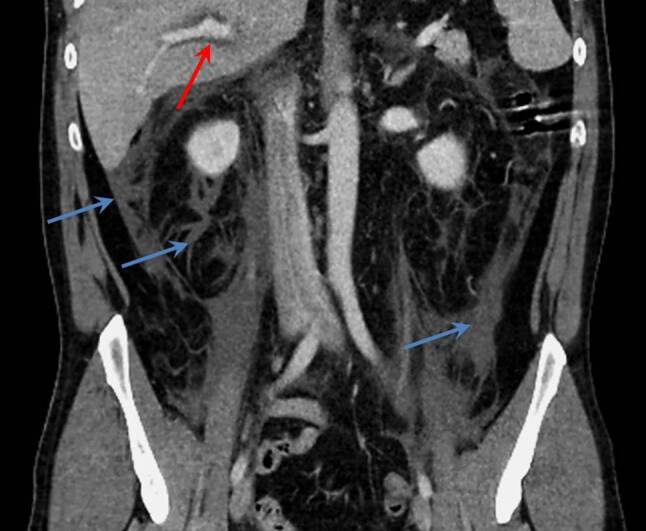
Contrast-enhanced abdominal CT scan in the coronal plane demonstrating mildly dilated intrahepatic bile ducts (*red arrow*) and retroperitoneal fluid collections (*blue arrows*)

**Fig. 2:**
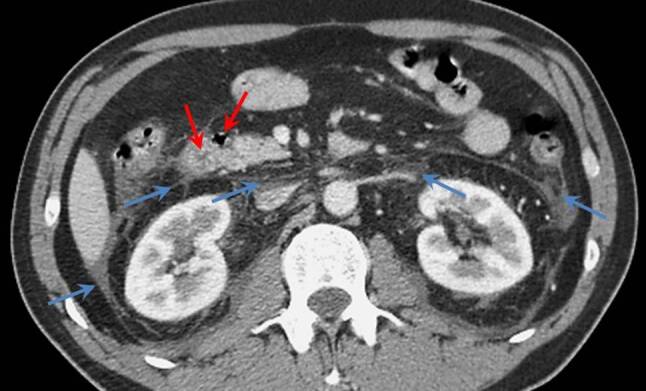
Contrast-enhanced abdominal CT scan in the axial plane demonstrating air-filled duodenal diverticulum with local mild duodenal wall thickening (*red arrows*) and fluid collections (*blue arrows*)

**Fig. 3:**
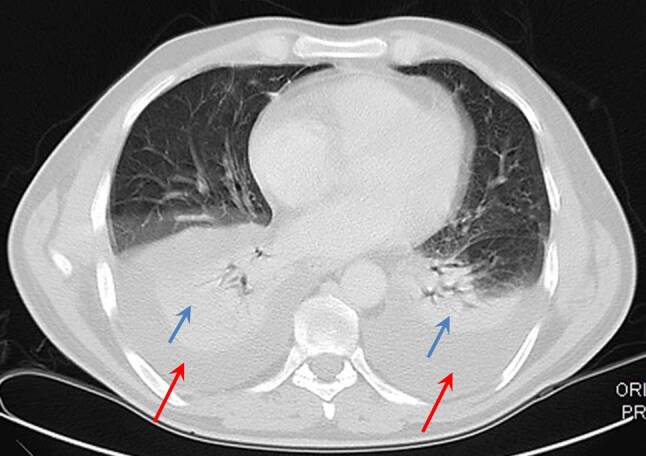
Chest CT scan in the axial plane and lung window revealing extensive bilateral pleural effusions (*red arrows*) with adjacent areas of pulmonary compression atelectasis (*blue arrows*)

A CT scan of the abdomen revealed mildly dilated intrahepatic bile ducts and moderate amounts of retroperitoneal fluid (Fig. 1), a thickened wall of the second portion of the duodenum and a peripapillary diverticulum (Fig. 2). Chest CT scan showed extensive bilateral pleural effusions with some compression atelectasis of the lower lobes (Fig. 3).

**Fig. 4:**
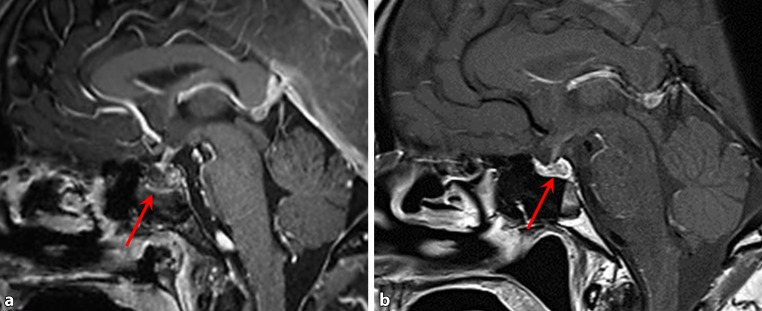
MRI of the brain in the sagittal plane showing reduced contrast enhancement of the enlarged pituitary gland with inhomogeneous contrast enhancement of the pituitary infundibulum (**a**, *red arrow*) and a reduced volume of the pituitary gland with persistent inhomogeneous enhancement after two months (**b**, *red arrow*)

The MRI of the brain demonstrated reduced contrast enhancement of the enlarged pituitary gland with inhomogeneous contrast enhancement of the pituitary infundibulum (Fig. 4a). A follow-up MRI after two months showed a reduced volume of the pituitary gland with persistent inhomogeneous enhancement (Fig. 4b).

### *Dr. M.-L. Aumüller*:

The reason for the development of delirium with dystonia, hyperkinetic movements (hemiballism) and photophobia in the discussed patient was initially unclear because the CT of the brain, electroencephalogram and laboratory analysis of the cerebrospinal fluid were all unremarkable. Since MRI of the cerebellum suggested apoplexy of the pituitary, macroadenoma or possible hypophysitis, an endocrinological work-up was carried out. Hormone assays showed normal serum levels of the luteinizing hormone, prolactin and human growth hormone, but low levels of testosterone, TSH, fT3, fT4 and insulin-like growth factor 1. Adrenocorticotropic hormone (ACTH) stimulation test revealed secondary adrenal insufficiency, which strongly suggests a pre-existing insufficiency of the pituitary rather than an acute onset (because formerly healthy adrenal glands would have been able to compensate the ACTH stimulation test). The suspected Addisonian crisis was managed with high-dose intravenous hydrocortisone and thyroid hormone replacement.

Comprehensive microbiological tests of the cerebrospinal fluid, pharyngeal and tracheal swabs, and secretion of the intraoperatively placed abdominal drain were all negative, as were several blood and urine cultures; however, repeated serological tests finally revealed the suspected infection with Puumala virus. On further history taking, the patient reported that he had taken down an old barn three weeks before the onset of symptoms.

Follow-up MRI two months later showed a significant decrease in the size of the inhomogeneity of the anterior pituitary from 1.4 cm to 0.7 cm (Fig. 4). Thus, the observed alterations of the pituitary may have been due to a pre-existing adenoma and apoplexy in the context of sepsis or hypophysitis caused by a severe course of Puumala virus infection as described earlier [33].

The significantly increased hemoglobin level found in the discussed patient was most likely due to exsiccosis caused by increased fever-induced perspiration or capillary leak, which is frequently found in systemic infections and commonly occurs concomitantly with acute renal failure. An increase in capillary permeability to proteins leads to the loss of the protein-rich fluid from the intravascular to the interstitial space. Although typically occurring in sepsis, many other diseases can lead to a “sepsis-like” syndrome with manifestations of diffuse pitting edema, exudative serous cavity effusions, noncardiogenic pulmonary edema, hypotension, and in some cases hypovolemic shock with multiple organ failure. These include among others the idiopathic systemic capillary leak syndrome (Clarkson’s disease), engraftment syndrome, differentiation syndrome, ovarian hyperstimulation syndrome, hemophagocytic lymphohistiocytosis, viral hemorrhagic fevers, autoimmune diseases, snake bite envenomation, and ricin poisoning. Moreover, some drugs such as interleukins, monoclonal antibodies, and gemcitabine can also cause capillary leak syndrome [39].

In some patients, angioedema which may result in pseudoacute abdomen by the sudden swelling of intestinal mucosa should be thought of as a differential diagnosis of capillary leak syndrome [40, 41]. Recently, a case of very severe Puumala hantavirus infection with capillary leak and shock was successfully treated with icatibant, a bradykinin receptor antagonist [42]. Although the exact pathophysiologic mechanisms remain to be elucidated, pronounced complement activation, prolonged leukocytosis, extensive fibrinolysis, circulating histones, and impaired liver function may be the underlying mechanisms [43].

### *Dr. G.J. Krejs*:

Hypophysitis secondary to hantavirus infection is rare and only a few cases have been reported in the literature [20, 32, 33]. One of these publications [20] is about a patient who presented at the University Medical Center Graz about 12 years ago and whose case had been discussed in our clinical-pathological conference series number 118. This 41-year-old worker was admitted because of a 1-week history of fever, lumbar pain, increasing jaundice, shortness of breath and blurred vision. Laboratory data revealed thrombocytopenia, acute renal failure (nephropathia epidemica) and hypopituitarism caused by secondary hypophysitis. The patient was most likely infected with Puumala virus when he had cleaned an attic located in the southeast region of Austria one week prior to the onset of symptoms [20]. This region is endemic for the bank vole (*Clethrionomys glareolus*), which is the reservoir of Puumala virus in Austria [25].

As in the discussed patient, symptoms of hantavirus infection may also include pseudoacute abdomen. Causes for this condition were first summarized by Henry Bockus [1] but surgeons have described diseases which simulate the acute abdomen already 100 years ago [44]. Included are among some infectious diseases such as typhoid fever, leptospirosis, malaria or hantavirus infection, a variety of specific clinical states such as possible irritation of the peritoneal pain receptors by osmotic mechanisms or by acid-base disturbance in diabetic ketoacidosis [45], disturbed capillary and subsequent organ perfusion caused by vascular occlusion in sickle cell crisis [46], mucosal swelling in angioedema [40, 41], autonomic nerve dysfunction in acute intermittent porphyria [47], a direct oculo-abdominal reflex triggered via the trigeminal nerve and completed via a loop from the nuclei of the vagus nerve by way of the vagal visceral motor and visceral sensory branches in acute glaucoma [48], negative effects on intestinal motility [49], the intestinal neural web or smooth muscles caused by lead or increased levels of δ‑aminolevulinic in chronic lead poisoning [50, 51], spontaneous bacterial peritonitis and some other conditions listed in Table 1.

### *Dr. R. Krause*:

Hantavirus infection came to the attention of the world during the Korean war (1950–1953) when more than 3000 United Nation troops fell ill with Korean hemorrhagic fever, also known as hemorrhagic fever with renal syndrome, which is an acute febrile infection characterized by renal failure and hemorrhagic manifestations that vary from petechiae to severe internal bleeding [52]; however, hantavirus as the etiologic agent of this disease was only recognized in 1978, when the virus was discovered in the lungs of its natural reservoir, the striped field mouse (*Apodemus agrarius*) [53]. For the milder form of hemorrhagic fever with renal syndrome, nephropathia epidemica, Puumala virus was found in bank voles (*Clethrionomys glareolus*) [54]. To date over 28 hantaviruses that cause disease in humans ranging from acute renal failure to pulmonary edema and severe hemorrhagic illness have been identified around the world [52]. Hantaviruses are enveloped RNA viruses that form a separate genus within the *Bunyaviridae* family and are closely associated with a single rodent species, which results from the co-evolution of the virus and the host [55]. Infection of other animals, such as moose [56], red fox [57] or domestic cats and dogs [58, 59] is considered to be a spillover with a minor or nonexistent risk for human infection [52]. In Europe, more than 9000 cases of hemorrhagic fever with renal syndrome are reported annually. The most predominant among them is the Puumala virus infection. Within Europe, hantavirus infection mainly occurs in European Russia, Finland, Sweden, Belgium, Germany and to a lesser extent in Norway, France, Hungary and Austria [60–62]. The risk of contracting hantavirus from rodents is related to the closeness of contact to infected animals. As in the discussed patient, humans are usually infected via aerosolized rodent excreta when cleaning cellars, barns, sheds or summer cottages in autumn, especially when these spaces are poorly ventilated, when working with hay and crops during harvesting, or cutting wood inside dusty woodsheds [52]. Incubation time is 1–5 weeks. Key findings in the pathogenesis of hantavirus infection include increased vascular permeability of microvascular beds and acute thrombocytopenia [63, 64]. Depending on the serotype, hantavirus infection in humans can affect different vascular beds, such as the capillaries in renal medulla or the pulmonary capillaries, resulting in two clinical syndromes: hemorrhagic fever with renal syndrome and hantavirus cardiopulmonary syndrome (caused by Sin Nombre virus and Andes virus) [52]. The pathogenesis of hantavirus infection is complex and involves specific immune responses, platelet dysfunction and deregulation of the endothelial barrier function. Although the viral antigen is present in several organs, infection mainly occurs in pulmonary or renal endothelial cells and macrophages [65, 66]. The virus replicates within the vascular endothelium but does not cause any direct cytopathic effects [67, 68] until inflammatory cell infiltration leads to tissue injury [52, 69, 70]. In the kidneys, the peritubular area of the distal nephron seems to be the main site of infection [69] causing tubular damage, increased glomerular permeability and subsequent massive proteinuria [70]. At present, no specific treatment is available for the treatment of hantavirus infection; the management is primarily supportive.

Physicians should highly suspect hantavirus infection when a patient presents with fever, acute renal failure, thrombocytopenia, pseudoacute abdomen and photophobia as was seen in the discussed case.

## Final diagnosis

Puumala-(hanta)virus infection
